# Consensus design of a calibration experiment for human fear conditioning

**DOI:** 10.1016/j.neubiorev.2023.105146

**Published:** 2023-05

**Authors:** Dominik R. Bach, Juliana Sporrer, Rany Abend, Tom Beckers, Joseph E. Dunsmoor, Miquel A. Fullana, Matthias Gamer, Dylan G. Gee, Alfons Hamm, Catherine A. Hartley, Ryan J. Herringa, Tanja Jovanovic, Raffael Kalisch, David C. Knight, Shmuel Lissek, Tina B. Lonsdorf, Christian J. Merz, Mohammed Milad, Jayne Morriss, Elizabeth A. Phelps, Daniel S. Pine, Andreas Olsson, Carien M. van Reekum, Daniela Schiller

**Affiliations:** a Wellcome Centre for Human Neuroimaging and Max Planck UCL Centre for Computational Psychiatry and Ageing Research, University College London, United Kingdom; b Hertz Chair for Artificial Intelligence and Neuroscience, Transdisciplinary Research Area “Life & Health”, University of Bonn, Germany; c National Institute of Mental Health Intramural Research Program, Bethesda, MD, USA; d KU Leuven, Faculty of Psychology and Educational Sciences/Leuven Brain Institute, Leuven, Belgium; e Department of Psychiatry and Behavioral Sciences, University of Texas at Austin, USA; f Institut d′Investigacions Biomèdiques August Pi i Sunyer (IDIBAPS), CIBERSAM, & Adult Psychiatry and Psychology Department, Institute of Neurosciences, Hospital Clínic, Barcelona, Spain; g Julius-Maximilians-University of Würzburg, Department of Psychology, Würzburg, Germany; h Yale University, Department of Psychology, New Haven, CT, USA; i Department of Psychology, University of Greifswald, Germany; j New York University, Department of Psychology, New York, NY, USA; k Department of Psychiatry, University of Wisconsin School of Medicine & Public Health, USA; l Department of Psychiatry and Behavioral Neurosciences, Wayne State University, Detroit, MI, USA; m Neuroimaging Center (NIC), Focus Program Translational Neuroscience (FTN), Johannes Gutenberg University Medical Center, Mainz, Germany; and Leibniz Institute for Resilience Research (LIR), Mainz, Germany; n University of Alabama at Birmingham, Department of Psychology, Birmingham, AL, USA; o Clinical Science and Psychopathology Research Program, Department of Psychology, University of Minnesota, Minneapolis, MN, USA; p University Medical Center Hamburg-Eppendorf, Institute of Systems Neuroscience, Hamburg, Germany; q Ruhr University Bochum, Faculty of Psychology, Institute of Cognitive Neuroscience, Department of Cognitive Psychology, Bochum, Germany; r Department of Psychiatry and Neuroscience Institute, NYU Grossman School of Medicine, New York, NY, USA; s School of Psychology and Clinical Language Sciences, University of Reading, United Kingdom; t Department of Psychology, Harvard University, 52 Oxford St., Cambridge, MA, USA; u Karolinska Institutet, Department of Clinical Neuroscience, Division of Psychology, Stockholm, Sweden; v Friedman Brain Institute, Department of Neuroscience, Department of Psychiatry, Icahn School of Medicine at Mt. Sinai, New York, NY, USA; w The Nathan S. Kline Institute for Psychiatric Research, Orangeburg, NY, USA

**Keywords:** Human fear conditioning, Experiment-based calibration, Multi-laboratory consensus, Calibration design, Measurement theory, Metrology, Experimental design

## Abstract

Fear conditioning is a widely used laboratory model to investigate learning, memory, and psychopathology across species. The quantification of learning in this paradigm is heterogeneous in humans and psychometric properties of different quantification methods can be difficult to establish. To overcome this obstacle, calibration is a standard metrological procedure in which well-defined values of a latent variable are generated in an established experimental paradigm. These intended values then serve as validity criterion to rank methods. Here, we develop a calibration protocol for human fear conditioning. Based on a literature review, series of workshops, and survey of N = 96 experts, we propose a calibration experiment and settings for 25 design variables to calibrate the measurement of fear conditioning. Design variables were chosen to be as theory-free as possible and allow wide applicability in different experimental contexts. Besides establishing a specific calibration procedure, the general calibration process we outline may serve as a blueprint for calibration efforts in other subfields of behavioral neuroscience that need measurement refinement.

## Highlights

• Heterogeneity in human fear conditioning readouts limits comparability of results.
• We report a multi-laboratory consensus on evaluation of readouts.
• Literature review identifies crucial design elements for calibration experiments.
• 25 design elements are formulated based on expert survey.
• Generic consensus process is translatable to other disciplines.

## 1 Introduction

Psychological theories are often formulated at the level of latent, not directly observable, variables. A case in point is the set of associative learning theories and relatedly reinforcement learning theory, which describe learning in terms of associative strengths or outcome predictions (Alonso and Schmajuk, 2012, Dickinson, 2009) and encompass fear conditioning (also termed threat conditioning, see Glossary) as a special case (Vervliet and Boddez, 2020). Of note, fear conditioning is also clinically relevant as an experimental model of stress-related and anxiety disorders (Beckers et al., 2023, Dunsmoor et al., 2015, Foa et al., 1989, Pittig et al., 2021). This underlines a further need for empirical tests and subsequent refinement of fear learning theories.

Empirical tests of psychological theories commonly rest on experiments in which the latent variable is manipulated by an independent variable, and its value is then inferred via its behavioral (including questionnaire responses) or physiological expression. This inference procedure is commonly termed measurement (see Glossary) (Estler, 1999). In the realm of human fear conditioning, measurement is often based on conditioned physiological changes (e.g., in skin conductance, pupil size, heart rate, or startle reflex), or explicit (often verbal) reports (e.g., ratings of fear, distress, or expectancy) (Lonsdorf et al., 2017).

Ideally, the measurement of a latent variable ought to have low error (Rigdon et al., 2020): it should be truthful (i.e., have low bias, see Glossary) and precise (i.e., have low variation) (BIPM, 2012). However, these desiderata are not accessible since the latent variable itself is not directly observable. Psychometric theory therefore recommends evaluating validity (AERA, 2014, Kane, 2016, Messick, 1987), and an important piece of validity evidence is the assessment of quantitative metrics (AERA, 2014) such as convergent validity and reliability (McDonald, 2013). In experimental practice, these metrics can be difficult to apply. While used to motivate some observables in fear conditioning research (Boddez et al., 2013, Scheveneels et al., 2016), convergent validity cannot adjudicate between two convergent measurement methods. Assessment of reliability, on the other hand, requires true scores to be relatively stable. Because expectation of the unconditioned stimulus (US) changes from trial to trial, assessment of retest-reliability in fear conditioning research typically requires the assumption of a stable individual propensity to acquire fear (Fredrikson et al., 1993, Torrents-Rodas et al., 2014, Zeidan et al., 2012), and, like split-half reliability, can only be approximately estimated by aggregating over many trials (Fredrikson et al., 1993).

Indeed, measurement recommendations in the field of fear conditioning are more often based on practical considerations and heuristic arguments than on psychometric properties, both regarding the type of observables and their transformations (see Glossary) (Ojala and Bach, 2020). These arguments, however, might be biased in favor of more traditional over novel measurement methods. Also, community consensus may sometimes be difficult to establish, as evidenced by rather broad guidelines (Blumenthal et al., 2005, Boucsein et al., 2012) and heterogeneous measurement practice across many fields of psychology (Simmons, Nelson, and Simonsohn, 2011) including fear conditioning research (Lonsdorf et al., 2019, Ney et al., 2018).

**Fig. 1:**
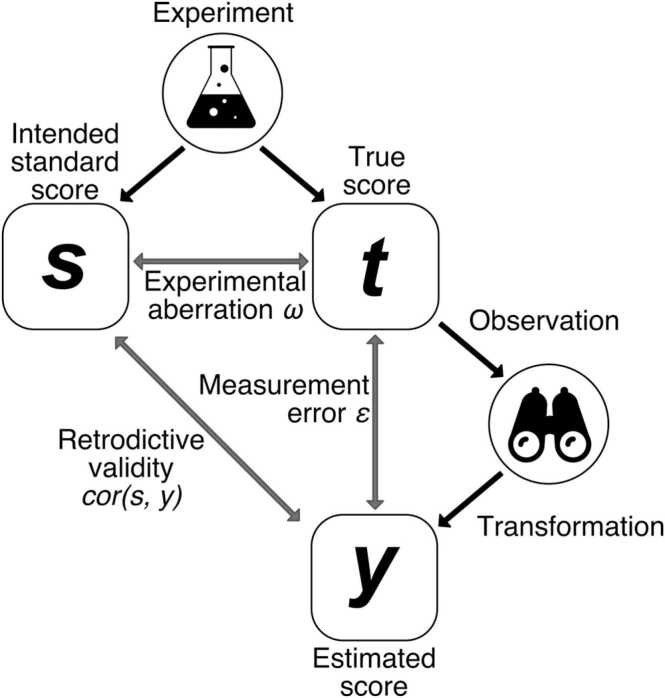
Illustration of the calibration approach. An established experimental procedure is used to generate true values of a latent attribute, which are measured with some measurement error. Ranking measurements by their retrodictive validity corresponds to their ranking by measurement error.

To evaluate a measurement and its associated error, a standard approach in natural sciences and technology builds on established procedures that are believed to create well-defined standard values of the quantity that one seeks to measure. This is commonly termed calibration (Phillips et al., 2001). A similar strategy can be used for evaluating psychological measurement, by harnessing experimental manipulations already agreed to have an effect on the latent variable (Bach et al., 2020). For example, fear conditioning is widely thought to instill high US expectation for a contingently coupled conditioned stimulus (CS+) and lower US expectation for a CS never coupled with US (CS-). The correlation between predicted and measured values of the latent variable (Fig. 1), termed retrodictive validity (Bach and Melinscak, 2020), can be used to evaluate measurement error (Bach et al., 2020). In an experiment, the true values of the latent variable will always deviate from the predicted values - this deviation is termed experimental aberration (Bach et al., 2020). For example, true US expectation will differ between persons. In turn, the measured values will deviate from the true values – this is termed measurement error (Bach et al., 2020). Aberration and measurement error jointly influence retrodictive validity. However, when comparing several measurement methods concurrently in the same experiment, the aberration is the same for all of them; the only thing that differs among them is their measurement error. Under fairly general statistical assumptions, ranking different measurement methods by their retrodictive validity (established within the same experiment) corresponds to their ranking by measurement error (Bach et al., 2020). Thus, comparing different measurement methods in the same calibration experiment, even in the presence of large aberration, identifies the method with minimal measurement error (Bach et al., 2020). The ordering of measurement methods according to their error, once established, generalizes - in theory - to different experimental circumstances, as long as the expression of the latent variable does not change.

To illustrate these concepts in a concrete example, consider recording skin conductance and pupil size in a fear conditioning experiment. According to most associative learning theories, both observables will be influenced by the presentation of the CS+ . Different methods of pre-processing these data time series (e.g., peak detection, outlier rejection, data transformation) result in different measured values of the US expectation. These different measured values can have different error (because they may be differently appropriate), and one may be interested in identifying the one(s) with the smallest error. In a calibration experiment, different observables such as skin conductance and pupil size, or different transformations of the same observable, can be compared by their retrodictive validity. The measurement method with highest retrodictive validity in this calibration experiment will have the lowest measurement error. It can be expected to also have the lowest measurement error in a different experiment, in which US expectation is manipulated by a different procedure, for example, fear conditioning followed by a particular form of fear extinction training. This occurs because US expectation can be assumed to be expressed in the same observable in the same way, independent of how the latent variable came about. However, if for example the CS/US interval is markedly increased, then participants might express the US expectation differently (perhaps at a later time point after CS onset), and then the best measurement method from the previous calibration might not be the best for this situation. Conditions that influence how the latent variable is expressed are termed "validity conditions" in calibration nomenclature (Phillips et al., 2001).

To introduce terminology and procedures on a more abstract level, Fig. 1 illustrates the general approach. First, an established experimental manipulation comes with intended values *s* (for standard) of a psychological attribute, for example CS+ and CS- result in high and low US expectation. We define true values *t* (the true US expectation in the example), which exist independently from the measurement procedure. (Notably, this "realist" definition is different from an "empiricist" definition in classical test theory where true scores are the expected value of the measurement, and are thus different for each measurement procedure (McDonald, 2013)). True scores will deviate from the intended values by some experimental aberration *ω* (which, in our terminology, is independent of the measurement). Once the true values are internally generated, they can be measured by taking one or several observations (SCR and pupil size in the example) and suitably transforming them into a measured score *y.* In the example, *y*_*1*_, *y*_*2*_, etc. can refer to the different observables or to different transformations (see Glossary). Each of these measured values (*y*_*1*_*, y*_*2*_,...) will deviate from *t* by some measurement error ε_1_, ε_2_, … which is specific to a particular measurement. The correlation between the intended standard values *s* and any measurement *y*_*i*_ is termed retrodictive validity. After the experiment, observations or transformations can now be compared by their retrodictive validity. Because *ω* is fixed, higher retrodictive validity implies smaller measurement error. It can be shown that measurement error in this procedure jointly depends on trueness of the measurement method, and its precision (Bach et al., 2020).

Calibration requires undisputed experimental manipulations of the latent variable. In science and technology, calibration is a community effort and institutionalized in large international bodies (BIPM, 2012). This is important to ensure a wide knowledge base and broad uptake of the resulting measures. Similar institutions do not yet exist in experimental psychology or behavioral neuroscience. The present paper describes an effort to establish a calibration experiment in a subfield of experimental psychology. The field of human fear conditioning appears particularly well-suited for this purpose for three reasons: first, considerable measurement heterogeneity exists, with at least 10 types of observables and various transformation methods for any of them, all used interchangeably to assess the same latent variable (Lonsdorf et al., 2019, Lonsdorf et al., 2017, Ojala and Bach, 2020). Secondly, despite theoretical disagreements about the nature of the underlying learning process (Dickinson, 2009, Houwer, 2011), consensus exists in the belief that the learning process results in learned expectation of the US, reflecting a latent variable that is then expressed in behavior or physiology. Third, improving measurement practice in fear conditioning research could have far-reaching clinical implications: fear conditioning is widely used as experimental model of psychopathology in anxiety and stress disorders (Beckers et al., 2023, Craske et al., 2014, Jacoby and Abramowitz, 2016, McNally, 2007), and various procedures aimed at clinical translation are being benchmarked in fear conditioning experiments, sometimes with limited replicability (Elsey et al., 2018, Kredlow et al., 2016). A calibration procedure for fear conditioning experiments could provide value for the field of associative learning theory, as well as a more robust route towards clinical improvements.

With this work, we aim to achieve two goals. First and foremost, we seek to develop a procedure that can be used for calibration in human fear conditioning research, with a particular focus on clinically-relevant experimental paradigms. This procedure should be usable to calibrate (and optimize) the different observables, as well as (possibly incremental) differences in data pre-processing and transformation methods for the same observable. We further seek to clearly define the applicability range of any results emanating from this calibration procedure, and to describe the procedure in a way that allows expanding this range by changing individual design variables, should particular research questions require different settings. Second, we hope that this serves a blueprint for a generic calibration process that could be applied in other fields of behavioral neuroscience.

## 2 Methods

**Fig. 2:**
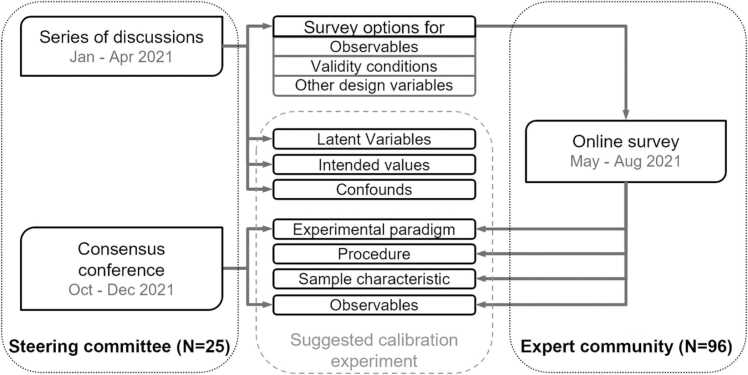
Illustration of the consensus process. Fundamental questions and survey options were decided in a steering committee, and then the wider expert community was surveyed about the validity conditions and other design variables. The calibration design was finalized by the steering committee.

In this section, we describe the process that was used for developing the proposed experimental design (Fig. 2), and then the design elements that had to be established.

### 2.1 Steering committee

Between November and December 2020, the first author contacted 34 human fear conditioning researchers across the world. Invitees were principal investigators who had published several human fear conditioning experiments over the past few years, selected to represent the diversity in the field with respect to methods, questions asked (methodology vs. associative learning theory vs. pre-clinical and clinical research), seniority, and world region. From those, 25 researchers decided to participate (4 non-tenured and 21 tenured faculty). In addition, 3 early-career researchers from the laboratories of the principal investigators joined. Of these 28 persons, 12 were based in US and 16 in (Western) Europe. Eleven researchers had conducted methodological investigations in the field of fear conditioning, and 12 had conducted clinical or pre-clinical research using fear conditioning paradigms. All 28 researchers participated in one or several of a series of discussions between January and April 2021, conducted as three live meetings via online platforms, email exchanges, and via collaborative documents. As a result of these discussions, we achieved consensus on the latent variables to be measured, their intended values, an initial list of confounds (see Glossary), and survey options for the experimental paradigm, the set of observables, and 22 other design variables.

### 2.2 Community survey

The goal of the community survey was two-fold. First, we sought to base the range of applicability of our procedure on common research designs, and to ensure high usability of the procedure. Thus, we sought to survey common experimental settings for the relevant design variables. Second, we sought to tap into the community’s practical experience with the impact of some design variables on variability of conditioned responses, on which there is currently relatively little methodological research. The anonymous online survey was implemented in RedCap (Harris et al., 2009) and advertised via different routes: among the steering committee's laboratories and professional networks, via a mailing list of fear conditioning researchers (N = 158), and finally we sought to achieve global coverage by directly identifying and approaching researchers that had published on the topic of human fear conditioning in the past years. Between May and October 2021, N = 96 individuals completed at least one section of the survey (13 postgraduate students, 29 postdocs, and 54 principal investigators). We deliberately did not solicit any personal or demographic information to minimize the possibility of identification of respondents. Median professional experience with fear conditioning experiments was reported as 8 years. To gauge scientific background, we asked for the (generally) "most common measure of human fear conditioning". Response options were "Autonomic responses" (N = 72), "Subjective experience of fear" (N = 22), "Gaze patterns" (N = 0) and "Overt behavior (such as freezing)" (N = 2). The survey and the survey results (excluding professional experience and free-text options, to avoid potential identification) are publicly available (https://osf.io/gsb2e/). Full data are available from the corresponding author under a data protection agreement.

### 2.3 Consensus conference

After the survey, the steering committee reconvened in October – December 2021 to reach consensus on all design elements of the calibration experiment.

### 2.4 Design elements

This section gives an abstract description of the design elements that we considered together with their theoretical underpinnings. The actual values and their justification are then presented in the results. This section may be used as a template for future calibration efforts in other fields of experimental psychology.

#### 2.4.1 Latent variable

This is the latent variable to be measured (in the initial example, this might be US expectation). Even if uncontroversial, it is important to explicitly ensure consensus such that design choices can unambiguously follow from this definition. To the extent that defining the latent variable requires in-depth discussion with exchange of theoretical arguments, it may be preferable to determine this in a consensus conference rather than subject it to a quantitative survey. For example, we found it instructive to clarify the distinction between "learned US expectation", "CS/US association", and "defensive state".

#### 2.4.2 Intended values

These are the values of the latent variable for the calibration experiment (in the initial example, "high vs. low" US expectation). At one extreme, the manipulation may be only established in the case of two values. At the other extreme, theory or experimental practice has already established a manipulation on a continuous scale (e.g., a percept of sound intensity (Fastl and Zwicker, 2007)). However, there may be theoretical or practical reasons to select a particular set of intended values even in this latter case. For example, reducing the number of intended values can enhance the precision of retrodictive validity estimates themselves (Bach, 2021). As another example from our field, associative strength may be positive or negative, but combining both directions into one experiment requires creating excitatory and inhibitory associations concurrently, which can be unwieldy.

#### 2.4.3 Confounds and inseparable attributes

These are other variables that covary with the latent variable in question, either because they are theoretically inseparable (e.g., they are thought to be distinctly generated but the expression of one variable is necessarily via the other), or because a particular experimental design renders them inseparable (experimental confounds). In our initial example, the physiological expression of US expectation may be mediated by general arousal, and this could be theoretically or experimentally inseparable.

#### 2.4.4 Experimental paradigm

This concerns the general structure of the calibration experiment and should be a procedure that is generally accepted to impact on the latent variable in question. Notably, the calibration approach rests on comparing two or more measurement methods within the same experiment. Thus, it only requires a positive – not necessarily high – correlation of intended values and true scores. In our example, a potential paradigm could be a simple fear conditioning task with one or two CSs, but also a more complex experiment in which multiple CSs are coupled with the US at different rates.

#### 2.4.5 Observables and transformations

Observables (e.g., behavioral choices, reaction times, skin conductance) are often called "measures" in empirical work. Here we use the term "observable" to distinguish from the "measured value", which is usually derived from the observable through some transformation, e.g., by outlier rejection, response quantification in time-series data, aggregation over trials, or estimation of measurement model parameters (Lonsdorf et al., 2017, Ojala and Bach, 2020). In our initial example, skin conductance responding may be derived from a peak-to-trough difference during time series pre-processing (Lonsdorf et al., 2017), but also parametrically estimated through a psychophysiological model (Bach and Melinscak, 2020). Importantly, such transformations can often be changed after the calibration experiment is performed, and hence existing calibration data can be used to test novel transformations. In contrast, observables always need to be determined beforehand, even if there is not (yet) consensus on which transformations to investigate later on. For example, if some researchers seek to benchmark a model-based measure of US expectation that combines several observables, then all need to be included, even if there is no universal agreement that they are useful on their own.

#### 2.4.6 Validity conditions

These are design variables that may influence how the latent variable or the identified confounds are expressed in observable behavior – i.e., that affect the measurement model. This will often include experimental timings – in our example, the time interval between CS and US (Castegnetti et al., 2017, Castegnetti et al., 2016, Redondo and Marcos, 2003) –, the inclusion of measurements that interact with each other or with the latent variable (Sjouwerman et al., 2016), and population characteristics (e.g., children vs. adults). Notably, the definition of validity conditions reflects the current state of the field and might change over time.

#### 2.4.7 Other design variables

The large set of other design variables in the chosen experimental paradigm can be thought of as broadly falling into two groups. The first are variables that influence experimental aberration. Here, it is preferable (although not mandatory, see (Bach et al., 2020)) to reduce systematic and random experimental aberration. In our initial example, one may argue that the CS/US association is less variable over participants if the set of CSs consists of easy-to-distinguish geometric shapes rather than musical chords. The second are variables that have no known impact on experimental aberration or measurement error. These variables may be chosen to maximize practicability of the calibration experiment. In our initial example, for pupil size it may not make a difference whether the inter-trial interval is 30 s or 10 s, but it may be more practical to keep it shorter.

## 3 Results

In the next section, we describe the results of the consensus process. We are generally concerned with cue conditioning paradigms in which an innocuous CS+ contingently co-terminates with an intrinsically aversive US, and a CS- does not (Lonsdorf et al., 2017). Since we are only concerned with fear conditioning, and to avoid cumbersome repetition, when we say "US", "conditioning" etc. we always mean "aversive US" and "learning to expect an aversive US".

### 3.1 Latent variable

In the field of fear conditioning, there are several possible latent variables to consider. We sought to select a variable that does not presuppose a particular theory of learning. Across learning theories, it is assumed that after contingent coupling of CS with US, presentation of the CS leads to an expectation of the US (which may or may not be consciously accessible). Our latent variable is therefore "learned US expectation". Although the term “expectation” is also used in the context of declarative knowledge, this terminological choice does not reflect commitment to any particular theory, or to any particular dependent variable. We chose the qualifier "learned" as a shorthand for “learned by direct experience” because there are other commonly used paradigms outside fear conditioning to generate a US expectation (e.g., by instruction (Mertens et al., 2018) or observation (Olsson et al., 2020)), and we sought to avoid the presumption that the ensuing US expectation is the same latent variable as in conditioning (although this may well be the case).^1^

Expecting a US is often thought to instill behavioral tendencies and physiological states, sometimes conceptualized as an "organismic state" or "defense system" (Hamm, 2020). Typical measures of fear conditioning (for example, derived from skin conductance) may in fact measure such states as intervening variables, rather than the US expectation itself. However, there is considerable theoretical disagreement about what exactly these states encompass, and how broad or selective they are. Hence, we did not select them as primary variables of interest. However, we note that if these states exist and if they are separate from the learning process, then they form theoretical confounds. Furthermore, behavioral tendencies and physiological responses appear to depend on the agents' goals and action options, which in turn depend on situational characteristics such as US predictability (Dunsmoor et al., 2007, Grady et al., 2016), US aversiveness (Dunsmoor et al., 2017), escape options (Low et al., 2015), CS/US belongingness (Ohman and Mineka, 2001), and the time left until the predicted US, i.e., the CS/US interval (Castegnetti et al., 2017, Castegnetti et al., 2016, Redondo and Marcos, 2003). Hence, changing these characteristics can change the way that US expectation is expressed in observables, and these form important validity conditions.

Among the steering committee, we identified a diversity of focus on different experimental phases (such as early and late fear acquisition training), with no obvious consensus on how precisely an experimental phase should be defined. To avoid arbitrary separation of experimental phases, we defined the latent variable of interest on a trial-by-trial level. This level of granularity allows researchers to analyze the calibration data in various ways, for example, on a trial-by-trial level as originally intended, but also collapsing across trials for different phases of the learning process.

Thus, the first latent variable is **trial-by-trial US expectation during fear acquisition training**. Next, there is a high clinical interest in evaluating manipulations that target previously learned US expectations (i.e., existing "US expectation memory"). This is usually measured in a separate experimental session without US delivery. Hence, our second latent variable is **US expectation memory recall.** Third, there is considerable interest in measuring the **reduction of trial-by-trial US expectation during fear extinction training**, which constitutes the third latent variable of interest**.**

From a neurobiological and clinical application perspective, it could be useful to assess recall and extinction after systems-level memory consolidation, i.e., after at least one night's sleep. From a practical perspective, a calibration experiment is much easier and cheaper to conduct if the recall test/fear extinction training session follows immediately after fear acquisition training. Although it is plausible that US expectation is expressed in the same way immediately or after consolidation, this is not known with certainty. We note that calibration experiments with immediate recall and recall after consolidation can otherwise be exactly the same, and the proposed calibration design can be used in both cases.

### 3.2 Intended values

Associative learning theory and clinical intervention research often treat US expectation and US expectation memory as continuous variables. Hence, it would be desirable to calibrate their measurement over several levels. However, the steering committee agreed that fear conditioning theory is not mature enough to allow uncontroversial definition of an experiment that generates more than two intended values on an interval scale; hence only two values will be used: High US expectation by contingently coupling a CS+ with a US, and low US expectation by never coupling a CS- with the US. Ideally, the latter case would lead to zero US expectation, but if CS+ and CS- are used in the same individuals and session, then generalization can elevate the US expectation of the CS- above zero. Hence we term these levels of US expectation "high" and "low". It would be desirable in the future to extend the calibration framework to more than two ordinal levels, or to advance learning theory to derive more than two interval-scale levels of US expectation.

### 3.3 Confounds

Many common fear conditioning measures target physiological states that can possibly be generated by other manipulations as well (Hamm, 2020). To the extent that such physiological states exist separately from the learning process, they form confounds that cannot be experimentally separated from the US expectation within a fear conditioning paradigm. This is an important caveat to consider when using novel (in particular pharmacological) experimental interventions outside the calibration. For example, suppose a drug affects how learning is expressed in the physiological state, but not learning itself. Then the observed absence of learning does not imply that learning did not take place. Such cases would be detectable with a memory recall test after drug wash-out. However, we are notably not aware of such specific experimental manipulations, and so we consider this confound as experimentally inseparable (at the moment).

Secondly, a duality of implicit and explicit learning processes has been suggested by some authors (McLaren et al., 2014). There is some circumstantial evidence that both might be expressed in the same measures (see (Ojala and Bach, 2020) for discussion), such that these two learning processes (if they exist) would be inseparable. A third potential confound is bottom-up attention elicited more by the CS+ than by the CS-, a process that some have in fact suggested to use as a measure of US expectation in spatial attention tasks (see (Ojala and Bach, 2020) for discussion). Both of these confounds might possibly be mitigated with learning paradigms that remove awareness of the CS, although we note there is considerable disagreement about suitable experimental paradigms (Lovibond and Shanks, 2002). Finally, an experimental, potentially separable confound, is the uncertainty of the US prediction. In the case of binary outcomes (US or no US), any metric of outcome uncertainty monotonically relates to how far away from 50% the US probability is (Bach and Dolan, 2012). Hence, this confound can be reduced by making reinforcement rates symmetric, i.e., having the same distance from 50% (although even in this case the actually learned probabilities might be asymmetric).

### 3.4 Experimental paradigm

Two basic paradigms are commonly used in fear conditioning research: single-cue and differential (sometimes also termed discriminant) conditioning. In the first procedure, one CS predicts a certain rate of US delivery. In non-human research, the response to the CS is often compared to baseline responding. In human research, habituation of conditioned responses (independent of reinforcement), and of learning-unrelated responses to the CS, necessitate using a control group in which no US is delivered. In the second, differential conditioning procedure, CS+ and CS- are used in the same individuals. In this procedure, it is sometimes assumed that the CS- is ignored and no learning takes place. Others have suggested that at least three learning processes take place: the CS+ comes to predict the US, the CS- is taken to also predict the US due to uncertainty or by generalization, and the CS- is then learned to predict the absence of the US (safety learning) (Kalisch et al., 2015). It is not known whether US expectation generated or modulated by generalization and safety learning are expressed in the same way as US expectation by CS/US coupling (although this may well be the case). Hence, the choice of a differential vs. single-cue learning paradigm forms a validity condition. We sought to use the paradigm that had the highest potential of being used in the community. In the survey, 94 out of 96 respondents stated they were most likely to use differential conditioning in their future research. From an experimental design perspective, we note that single-cue and differential conditioning procedures can be created from one another without changing (most of) the other design variables.

### 3.5 Design variables

**Fig. 3:**
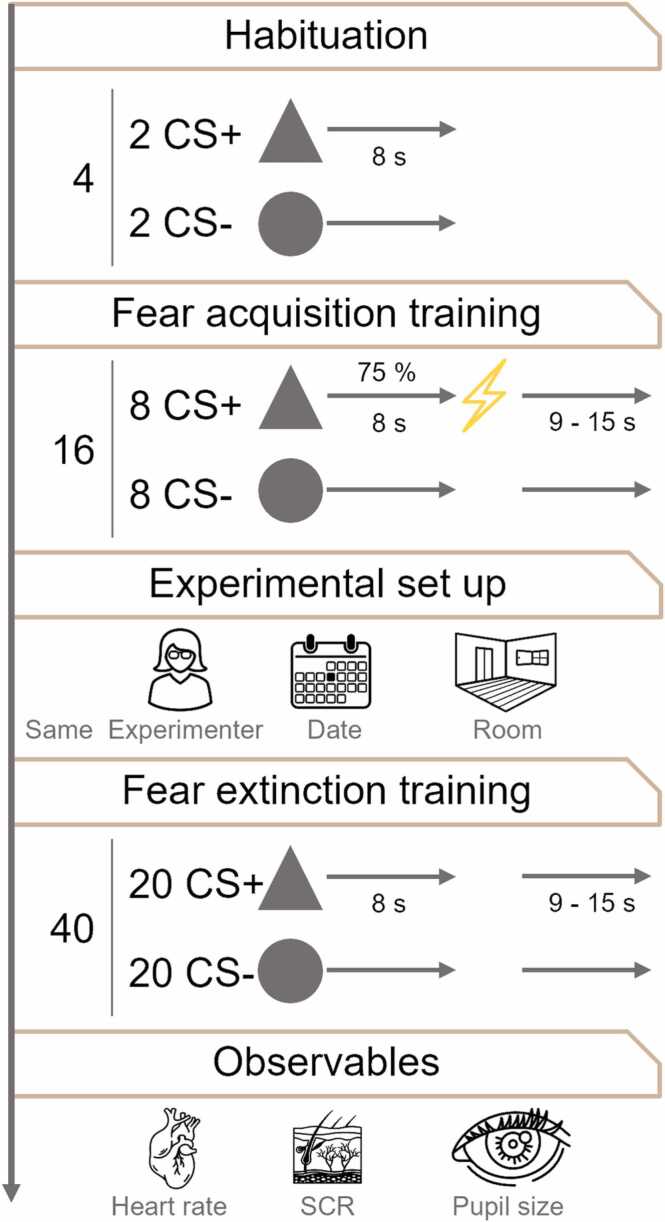
Illustration of the proposed calibration experiment.

The steering committee grouped design variables into three categories: validity conditions that may influence how the latent variable is expressed (i.e., the measurement model), design variables that may influence how much the latent variable varies over participants (i.e., the experimental aberration), and design variables that may not have any impact on either and can be chosen by practical considerations. Notably, this categorization is mostly based on heuristic arguments and experience; there is limited methodological research that directly addresses these questions. Table 1 lists the design variables by the logic of experimental design (see Fig. 3 for illustration), whereas in the text we group them by their status, give some arguments for their classification, and how we arrived at the suggested values.

**Table 1:** Suggested calibration experiment. Relevant design variables were selected and classified by the steering committee, and their most common (validity conditions) or most useful (others) value surveyed among N = 96 experts. Future experiments building on calibration results should maintain the validity conditions but can deviate in the other variables. See Fig. 3 for a visualization.

Design variable	Suggested value	Status	Decision basis
***1. Experimental paradigm***			
*Basic paradigm*	Differential fear conditioning	Validity condition	Field survey
*Intended values*	High (CS+), low (CS-) US expectation	Only relevant for calibration	Steering committee
*Total number of trials: habituation*	4 (2 CS+, 2 CS-)	Validity condition	Field survey
*Total number of trials: fear acquisition training*	16 (8 CS+, 8 CS-)	Validity condition for measurement of recall	Field survey
*Total number of trials: fear extinction training*	40 (20 CS+, 20 CS-)	Number of trials included in any measurement method is a validity condition for that measurement method only	Field survey
*Interval between fear acquisition and extinction training*	Minimal	Validity condition	Steering committee
*CS/US interval*	8 s	Validity condition	Field survey/Steering committee
*Inter-trial-interval*	9–15 s uniform	Validity condition	Field survey
*Incidental task*	No	Validity condition	Field survey
*Inclusion of attentional capture/disengagement tasks as observables*	No	Validity condition	Steering committee
*Type of task instructions*	"US may or may not occur sometimes after CS"	Validity condition	Field survey
*CS reinforcement rate*	CS+ : 75% CS-: never	Aberration	Field survey Steering committee
*CS*	Visual: simple shape and/or single color	Aberration	Field survey
*US*	Individually calibrated uncomfortable electric stimulus	Aberration	Field survey
*Trial order*	Constrained random (no more than 3 subsequent trials of the same type, first and last CS of each session counterbalanced between participants)	Aberration	Field survey
*CS+ and CS- number*	Equal	Aberration	Field survey
***2. Procedure***			
*Time of day*	No fixed time of day	(Potentially a validity condition if fixed)	Field survey
*Contextual consistency*	Same room, same experimenter (within-subjects)	Validity condition	Field survey
*Light conditions*	Regular	Aberration	Field survey
***3. Sample***			
*Age*	18–45 years as priority	Validity condition	Field survey
*Regular use of psychotropic substances (recreational/prescription; excluding alcohol and nicotine)*	Exclude for 3 months	Validity condition	Steering committee
*History of neuropsychiatric disorders including addiction (including alcohol but excluding nicotine)*	Strict exclusion	Validity condition	Field survey
*Previous participation in fear conditioning studies*	Exclude for 3 months	Validity condition	Field survey
***4. Observables***			
*Inclusion of startle probes*	No	Validity condition	Steering committee/Field survey
*Inclusion of explicit ratings*	Not before end of fear extinction training	Validity condition	Steering committee/Field survey
*Physiological observables (suggested minimum set)*	Skin conductance, heart rate, pupil size	Only relevant for calibration	Field survey

#### 3.5.1 Validity conditions

These are the design variables that potentially impact how the latent variable is expressed in behavior, i.e., the measurement model. The steering committee decided on inclusion of variables in this list by consensus. Because the calibration result will only be strictly valid within these conditions, we sought to set the values in a way that maximizes the usability of results – and so they are largely based on community demand, as indicated in the survey.

**Number of trials in habituation (pre-acquisition):** Presenting the CS (without reinforcement) before the fear acquisition training phase could reduce responses to the CS and thereby change the expression of US expectation, thus forming a validity condition. The community (N = 96) indicated they were most likely to use 0–100 trials in total, with a median of 4 trials (2 CS+, 2 CS-) across respondents.

**Number of trials in fear acquisition training:** There is a possibility that the expression of US expectation changes over trials (for example, skin conductance responses [SCR] appear to decay independently of the learning process). Hence, the number of trials in acquisition is a validity condition for the recall/fear extinction training session. The community (N = 96) indicated they were most likely to use 6–100 trials in total, with a median of 16 trials (8 CS+, 8 CS-) across respondents.

**Number of trials in fear extinction training:** The expression of previously learned US expectation changes over time due to extinction (Khemka et al., 2017), and the assessment of extinction itself may also change depending on the number of trials included. The numbers of trials used to assess US expectation memory recall, and to assess extinction, are therefore important validity conditions. For the calibration experiment, however, this is largely irrelevant as long as the experiment contains more trials than what is planned to be used in any recall or extinction measurement (because one can always restrict any measurement method to a subset of trials). The community (N = 96) indicated they were most likely to use 2–100 trials, with a median of 20 trials across respondents. The steering committee decided to include more than 20 trials to enable a rigorous assessment of extinction measurement, and pragmatically converged on 40 trials which includes the large majority of suggested trials in fear extinction training (88 out of 96 respondents, 92%).

**CS/US interval:** Conditioned responses are assumed to express US expectation, and the time point of their expression can vary according to the CS/US interval (Castegnetti et al., 2017, Castegnetti et al., 2016, Redondo and Marcos, 2003). Also, the CS/US interval influences scoring strategies for time-series data (e.g., SCR peak windows). This renders the CS/US interval a validity condition. The community (N = 82) indicated they were most likely to use 0–26 s, with a median of 6 s across respondents. In light of a community preference for a large number of reinforced trials (see below), the steering committee found consensus that a slightly longer CS/US interval of 8 s would facilitate analysis of the most popular observable (SCR) on reinforced trials. Hence, we suggest using a CS/US interval of 8 s

**Inter-trial interval:** Some popular conditioned responses such as SCR last much longer (up to 90 s decay to baseline, (Bach et al., 2010)) than a typical inter-trial interval. The ability to separate these responses, and thus the measurement error, depends to some extent on the inter-trial interval (Alexander et al., 2005, Barry et al., 1993). Hence, the inter-trial interval is a validity condition for all those observables in which responses outlast trial duration, in particular SCRs. The community (N = 82) indicated they were most likely to use an average inter-trial interval of 1–30 s, with a median of 12 s across respondents. The majority (N = 49, 60%) indicated using a variable inter-trial interval, with a median range of 6 s. The most commonly indicated distribution of inter-trial intervals was uniform. Thus, we suggest a 9–15 s uniform distribution of inter-trial intervals.

**Incidental task:** Incidental tasks such as CS detection or identification may influence the expression of some conditioned responses (e.g., SCRs (Boucsein, 2012)); hence this is a validity condition. As a special type of incidental task, it has been suggested to use attentional capture or disengagement tasks to measure US expectation (see for review (Ojala and Bach, 2020)). A priori, the steering committee found consensus that attentional measures of US expectation are not mature and widespread enough to include them into the calibration experiment. In terms of other incidental tasks, the majority (N = 67, 82%) of respondents (N = 82) indicated they were most likely to use no incidental task, which is consequently what we suggest here.

**Fig. 4:**
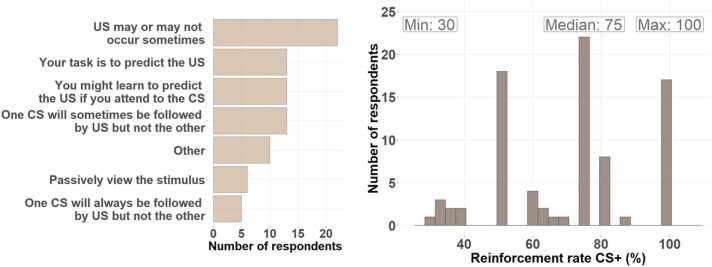
Preferred type of instructions (left) and reinforcement rate (right) was heterogeneous in the surveyed expert community.

**Task instructions:** Some commonly used instructions contain propositional information about the task structure (e.g., that only one CS will be reinforced). To the extent that this changes attention to the CS, which itself may influence some popular observables (e.g., SCR (Luck and Lipp, 2016; Mertens et al., 2018)), instructions may impact on the expression of the US expectation and therefore constitute a validity condition. Among the community (N = 82), there was no strong majority for any particular type of task instructions (see Fig. 4). N = 51 respondents (62%) would not include propositional information about the task structure (i.e., the fact that only one out of two CS will be reinforced) in the instructions. Also, N = 51 (62%) preferred instructions that guide attention towards the CS/US contingency. Among the choices provided, the most commonly selected task instructions were: "US may or may not occur sometimes" (N = 22; 27%). We suggest a type of instruction that combines the most popular instruction with a focus on CS/US contingencies, as the latter (also termed "general contingency instructions") has been shown to increase learning (Mertens et al., 2021): "US may or may not occur sometimes after the CS". The full suggested instructions are included as an appendix.

**Inclusion of explicit ratings between trials:** The inclusion of subjective ratings may change the learning process (Atlas et al., 2021), for example by favoring explicit over implicit learning (if these exist separately), and thus the latent variable. Because of physiological responses (e.g., SCRs) to the rating scales, their inclusion can also affect the measurement model for at least some observables. Therefore, this forms a validity condition. The steering committee decided a priori that the calibration of physiological measures and of subjective ratings should be performed in separate experiments. In the community survey (N = 90), N = 47 (52%) were likely or very likely to include trial-by-trial subjective ratings. This was far outnumbered by the number of respondents interested in the most popular physiological measure, namely, SCR (N = 81, 90%). We suggest not using subjective ratings until the end of the fear extinction training phase in the initial calibration experiment.

**Inclusion of startle probes:** Startle probes during CS presentation can influence the progress of learning (Sjouwerman et al., 2016) as well as elicit other physiological responses; their inclusion therefore forms an important validity condition. The steering committee decided a priori that the calibration of startle responses and of other physiological measures should be done in separate experiments. In the community survey (N = 90), N = 40 (44%) respondents were likely or very likely to include startle in fear acquisition training and N = 41 (46%) in recall/fear extinction training. This is in contrast with the aforementioned 90% who would use SCRs. We suggest not using startle probes in the initial calibration experiment.

**Time of day:** Time of day, if constrained, could be a validity condition under certain circumstances. For example, if the recall test is performed one day later, then the interval between fear acquisition training and sleep may influence the consolidation process, and possibly, the expression of US expectation memory on the next day. In the community survey (N = 82), the majority (N = 59, 72%) indicated that they would not fix or constrain the time of day; hence this is not a validity condition for the calibration procedure.

**Contextual consistency:** Context has a major impact on the expression of US expectation (Maren et al., 2013), but beyond categorical statements such as "same context", many practical subtleties may influence to what extent a participant perceives the context as similar or different; hence these practicalities form validity conditions. Out of N = 96 respondents, N = 76 (79%) would keep the room consistent, N = 56 (53%) the experimenter, N = 39 (40%) the time of day, and only N = 5 (5%) would keep none of these elements the same. For the calibration procedure, we suggest keeping room and experimenter the same between fear acquisition and recall/fear extinction training (but not necessarily between participants).

**Sample:** There is a suggestion that different populations may express US expectation differently (e.g., just by inherited or age-dependent differences in peripheral physiology (Shechner et al., 2014)). Hence, the sample forms a validity condition. The steering committee found consensus a priori to strictly exclude participants that regularly used prescription or recreational psychotropic substances (other than alcohol and nicotine) anytime during the 3 months before the experiment, and to strictly exclude anybody with a history of addictive disorder (other than nicotine addiction). The committee also agreed not to require recording of self-reported ethnicity, due to ethical and practical concerns associated with such procedure at least in some countries. The community (N = 73) indicated a large age range with lower limit at 8–19 years (median 18 years) and upper limit from 25 years up to unlimited age (median 45 years). Furthermore, the community indicated that they were slightly more likely to exclude participants with a history of neuropsychiatric disorder (43/82 respondents, 52%), than to include them under diagnostic screening. Finally, 47/81 respondents (58%) were likely to exclude persons who had previously participated in fear conditioning experiments. The interval after previous experiments during which to exclude participants varied from 1 month to forever, with a median of 3 months. For the calibration procedure, we suggest recruiting persons without neuropsychiatric history (including any addiction history other than nicotine addiction), who haven't participated in fear conditioning experiments or regularly used psychotropic substances (excluding alcohol or nicotine) for at least 3 months. We also suggest prioritizing recruitment of persons between 18 and 45 years of age, noting that if participants outside this age range are recruited it is easy to restrict or expand the sample for particular calibration purposes after the experiment. Once a calibration sample is defined, however, calibration is only meaningful if all participants that completed the calibration experiment per protocol are included in the calibration data, and no participants are excluded post-hoc.

#### 3.5.2 Variables influencing aberration

These are the design variables that may influence how consistently participants learn the US expectation, and thus the variability of the latent variable across participants. They are only relevant for the calibration experiment; further research building on this calibration can deviate from these settings. These variables do not strictly have to be optimized, but theoretical research suggests that minimizing aberration can increase the precision of retrodictive validity estimates themselves (Bach, 2021). In general, we instructed respondents explicitly to indicate what they thought would minimize variability between participants, even if this was not the design they would normally use for their own substantive research.

**CS+ reinforcement rate:** This may influence the progress of learning, and potentially the variability between participants. The community (N = 82) suggested 30%− 100% (median 75%) would minimize variability (see Fig. 4). We suggest a 75% reinforcement rate. Because this may necessitate analyzing reinforced CS+ trials, we also suggest using a long CS/US interval of 8 s (see above). We note that the reinforcement rates suggested for CS+ and CS- imply different outcome uncertainty, which thus forms an important experimental confound.

**CS:** Some types of CS might be easier to identify and distinguish than others, and for some there may be more variability between participants. The community (N = 77) suggested to use visual CS (N = 61, 79%) rather than auditory/tactile/olfactory CS, artificial (N = 47, 61%) rather than natural CS, neutral (N = 58, 75%) rather than salient or relevant CS, and perceptually simple (N = 68, 88%) rather than complex CS. Within the category of visual, artificial, neutral, and simple CS, N = 27 respondents (35%) suggested shape as discriminant stimulus dimension, N = 21 suggested color (27%), and N = 27 (35%) thought it made no difference. We suggest using two simple geometric shapes, and/or two monochrome colors. Since we suggest including pupillometry as observable (see below), we suggest isoluminant colors for both CS and background, and small CSs (e.g., below 5° visual angle) or an isoluminant fixation cross at CS center. We note that CS type may also constitute a validity condition for some specific conditioned responses (Holland, 1977), although not for the observables suggested here.

**US:** Some types of US might lead to more consistent learning than others. The community (N = 96) indicated that individually calibrated electric shock was among the US types that would minimize variability between participants (N = 56, 58%); the next frequently selected US type was a human scream (N = 19, 20%). We suggest using individually calibrated electric shock as US type. We note that US type may also constitute a validity condition for some specific conditioned responses (Jenkins and Moore, 1973), although not for the observables suggested here.

**Trial order:** The community (N = 89) suggested to minimize variability between participants by using fixed trial order (N = 12, 13%), counterbalanced trial order (N = 15, 17%), constrained random trial order (N = 44, 49%), or that it makes no difference (N = 6, 7%). In a free-text field, the most commonly named constraint was that no more than 2–4 trials of the same type should follow in a row. Another frequently named constraint was to fix or counterbalance the first and the last trial in terms of CS and reinforcement. We suggest using constrained random trial order with no more than 3 trials of the same type in a row and counterbalancing the type of first and last trial between participants.

**Light conditions:** Some physiological responses are altered in darkness (e.g., darkness-potentiated startle). The community (N = 77) suggested to use regular lighting conditions (N = 30, 39%), darkness (N = 26, 34%), or that it made no difference (N = 21, 27%). We suggest using regular lighting.

### 3.6 Observables

Only observables included in the calibration experiment can later be used for evaluation of new data transformations. The community indicated they were likely or very likely to base the measurement of US expectation on SCRs (81/90 respondents, 90%), eye tracking measures such as pupil size or gaze direction (36/89 respondents, 40%), heart rhythm measures (42/89 respondents, 47%), respiration measures (9/89 respondents, 10%), and blood pressure (3/89 respondents, 3%). To the extent that the equipment is available, we suggest recording skin conductance, pupil size, and heart rate (by ECG or pulse measurement) as a minimum set of observables for the calibration experiment.

## 4 Discussion

In this paper, we report a consensus process to establish a calibration experiment for fear conditioning, with N = 28 experts in the steering committee and N = 96 anonymous participants, probably including part of the steering committee, in an online survey in the field. With this report, we hope to achieve two things. First, we provide a generic blueprint for future calibration efforts of latent variables concerning other psychological processes. These include declarative memory, spatial attention, decision confidence, and many others. Second, we propose a specific design for a generally accepted calibration procedure in the field of fear conditioning.

On the first goal, we highlight the value of constructive in-depth discussions among experts. We found that the diverse expertise of the discussants often revealed aspects that not everybody had considered before. Also, at least in our field, terminology and research practice are heterogeneous (Lonsdorf et al., 2019, Lonsdorf et al., 2017, Ojala and Bach, 2020), and many differences and commonalities could be clarified in these qualitative discussions. Overall, they strongly shaped the design of the community survey. Our process closely resembles a recent attempt to establish theoretical benchmarks for working memory – empirical phenomena that any theory should explain (Oberauer et al., 2018) – with similar order of the number of participants, both in the initial discussions and the expert survey (N = 81 as compared to N = 96 here). As a general problem, there was a strong bias towards principal investigators in the survey, and the number of early-career respondents was even smaller than the community of laboratory members of the steering committee. It may not be clear to early-career researchers why their opinion is valuable here when they are less experienced, or why input in such calibration process might be beneficial for them when the end result – fully calibrated measures – is likely to take several years, possibly beyond the time horizon of today's early career trajectories. It might be possible that connecting the calibration process more strongly with professional and scientific societies, and disseminating the underlying principles on conferences outside the field of psychological methods, could encourage larger community uptake and stronger involvement of early-career researchers.

On the second goal, we discovered several limitations in the state of knowledge of our field. Specifically, many variables are suspected to be validity conditions or influencing aberration, but despite high-level reviews on these issues (Lonsdorf et al., 2019, Lonsdorf et al., 2017, Ojala and Bach, 2020), there is little focused experimental research to address this. Our arguments are therefore largely heuristic and based on experience but not systematic empirical investigation – precisely the kind of arguments we would have liked to avoid. Nevertheless, the suggested calibration approach provides for straightforward (if resource-intensive) empirical tests of these heuristic arguments. The calibration design can easily be modified to accommodate and compare different values of these variables in a between-subjects design. To verify validity conditions in such experiments, there are two approaches. First, one can directly compare the expression of the latent variable in two samples. For example, one may find that the peak time of conditioned SCRs is different between children and adults, corresponding to a different expression of learned US expectation. This would verify age range as a validity condition (or not). Secondly, one can just compare the ranking of measurement methods by retrodictive validity in two different groups with different settings of the design variable, for example between children and adults. This may find that different peak windows for SCR scoring are optimal in these two groups, and again verify age as a validity condition (or not). However, this latter black-box approach might miss out on subtle differences not captured in current measurement methods (for example, the specific SCR time course in children might not be captured in any current measurement model) and is also equivalent to simply performing calibration separately for the two age groups. To verify aberration variables, one can compare retrodictive validity of the same measurement method between two values of the design variable (e.g. (Sjouwerman et al., 2016)). This amounts to empirical design optimization (Melinscak and Bach, 2020) and may also be helpful to establish robust experimental designs outside the calibration context. All of these approaches require large enough sample sizes. We believe that ultimately it will be possible to delineate how well calibration results generalize or not, and what the most important validity conditions are. This approach also fosters standardization across groups, facilitating multi-site calibration studies. More generally, results could also be used to calibrate other important latent variables in our field, such as instructed and observed US expectation, learned US avoidance, or reconsolidation.

Sample size for this (or any) calibration experiment should enable distinguishing retrodictive validity estimates at the desired level of resolution. However, determining the appropriate sample size a priori is not trivial, because the variance of the retrodictive validity estimator does not only depend on the variance of the underlying measured values, but also on their distribution, which will often be non-normal by design (Bach, 2021). Thus, we suggest conducting an initial calibration experiment in a large sample of pragmatically chosen size (e.g. N = 100) and assessing variability via bootstrapping. This might then give an indication of whether the sample size was appropriate to distinguish retrodictive validity of common measurement methods, and will suggest sample sizes for follow-up calibration studies.

In general, we believe that the calibration approach advocated here might benefit many fields of behavioral sciences. Starting with the most closely related concepts, it might be necessary to alter certain validity conditions in a substantive experiment for a particular question in human fear conditioning research. For example, one may be interested in US expectancy memory after overnight consolidation. A suitable calibration experiment for such cases can be directly derived from the consensus design reported here, by altering just a small number of design variables and keeping the others constant. Somewhat further away, there might be an interest in calibrating methods for rodent fear conditioning. This could be based on the conceptual considerations reported here, including the latent variable and many, albeit not all, design variables, while some design variables would need to be removed (e.g. neuropsychiatric history), changed (e.g. definition of drug naivety), or added (e.g. strain). In addition, most of the values of the design variables would likely be changed, ideally based on another consensus process. Finally, the measurement of any psychological or cognitive attribute might be calibrated in the same way, with the caveat that consensus on at least some experimental procedures and latent variables needs to exist in the first place. To put this prerequisite into the terminology of null hypothesis significance tests: calibration assumes – based on previous substantive research – that the null hypothesis is already rejected, and identifies the measurement method that generates most evidence against it. If there is no strong agreement on empirical phenomena, latent variables, and suitable experimental procedures, then the calibration approach could lead to a dangerous conflation of methods research with hypothesis testing. Thus, it could indirectly contribute to unfavorable research practices such as p-hacking and harking (Simmons et al., 2011). Clearly, psychology subfields with a prevalence of strong (falsifiable) theories will find it easier to adopt this approach than those in which even basic experimental phenomena are contested (Oberauer and Lewandowsky, 2019). However, even within consolidated fields, there can be a tendency to overinterpret observables (Houwer, 2011). In the present work, we have deliberately attempted to stay clear of theoretical debates and disagreements, rather than push a specific theoretical view. We note that many measurement methods make implicit or explicit assumptions. We advocate making these assumptions transparent, at best formalizing them in measurement models such as psychophysiological models (Bach et al., 2018, Bach and Friston, 2013, Bach and Melinscak, 2020), and testing them experimentally. Ideally, then, measurement methods that make the most appropriate assumptions will also turn out to have the most favorable metrological properties.

To summarize, in this manuscript we attempted to design a calibration procedure for the measurement of human fear conditioning. With this work, we hope to have contributed to the improvement of measurement methods within our own field, and across behavioral neuroscience and psychology.

## Funding sources

DRB receives funding from the 10.13039/501100000781European Research Council (ERC) under the European Union’s Horizon 2020 research and innovation programme (ERC-2018 CoG-816564 ActionContraThreat), and from the 10.13039/501100000272National Institute for Health Research (NIHR) UCLH Biomedical Research Centre. The Wellcome Centre for Human Neuroimaging is supported by core funding from the Wellcome (203147/Z/16/Z). RA is supported by the Brain & Behavior Research Foundation (28239). MAF receives funding from the Instituto de Salud Carlos II co-funded by the 10.13039/501100000780European Union (PI 19/00272). TJ receives funding from the National Institutes for Health (R01 MH111682) and 10.13039/100000874Brain and Behavior Research Foundation. CJM receives funding from the 10.13039/501100001659Deutsche Forschungsgemeinschaft (DFG; German Research Foundation) within the SFB 1280 Extinction Learning (grant number 316803389 - SFB1280; project A09). TBL receives funding from the 10.13039/501100001659German Research Foundation (DFG LO1980/4-1, LO1980/7-1, LO1980/10-1). DSP is supported by NIMH-IRP Project ZIA-MH002781. DS is supported by 10.13039/100000002NIH (R01MH122611, R01MH123069).

## Author contributions

DRB conceptualized the research, invited the collaborators, analyzed field survey results, and wrote the first draft of the manuscript. JS prepared and conducted the field survey. All authors contributed to the design of the survey, the formal analysis and to the manuscript.

## Figures and Tables

**Table:** 

**Aberration**	Deviation of the true values of the latent variable from what was predicted or intended (for example, due to random or systematic variation between participants). We define the true values as independent of the measurement procedure ("realist" definition). Hence, aberration does not depend on which measurement method is used. Aberration can be systematic (i.e., bias from intended values) or random.
**Classical conditioning**	Modification of a response to a conditioned stimulus (CS) that can be attributed to repeated exposures to contingent presentations of CS and unconditioned stimulus (US).
**Confound**	A latent variable that correlates with the investigated latent variable, and might have a separate influence on the measurement.
**Fear conditioning** ***Also termed: Threat conditioning, Aversive Pavlovian conditioning***	Classical conditioning with a defense-relevant US. The term "fear" conditioning has been challenged (LeDoux, 2014) due to the connotation of "fear" with feelings and conscious experience. Here we stick with this term for simplicity, reflecting its widespread usage in learning psychology and biology, but noting that a relevant proportion of the authors advocates alternative terminology.
**Measurement**	We use the term measurement to refer to the quantification of a latent variable in an experiment. Measurement encompasses observation and transformation (see below).
**Measurement error**	Deviation of the measured values of the latent variable from the true values. Measurement error depends on the measurement. Measurement error can be systematic (i.e., bias from the true scores) or random. This distinction in the context of calibration is discussed in (Bach et al., 2020). In classical test theory, measurement error often only refers to random error, whereas the true value is defined to have no bias.
**Measurement model**	Formal specification of how a latent variable relates to observables. This can be a very simple model as in classical test theory (which is often implicitly used in operationalization approaches), or include non-linear relationships between different latent variables and observables as in drift-diffusion models or psychophysiological models. Structural equation modelling often combines a measurement model with a model of how latent variables relate to each other (the latent process).
**Observation**	A data type taken in an experiment, for example, reaction times, skin conductance, or questionnaire responses.
**Precision**	A measurement is precise if it has small variation under constant true scores. One way of assessing precision is to assume constant true scores and assess reliability.
**Transformation**	We use the term transformation to refer to the procedure of quantifying a latent variable from an existing observable. This can be based on operationalization, on ad-hoc procedures such as outlier detection, aggregation over multiple trials, detection of peaks in a time series, or on a formal measurement model (see above). Some approaches can include the integration of multiple observations of the same or different types into one measurement.
**Trueness**	A measurement is truthful if it is, on average, close to the true score of the latent variable. Trueness is the opposite of bias. This is related to the psychometric concept of validity – a valid measure would have high trueness.
**Validity condition**	Experimental design feature that influences how a latent variable is expressed in behavior or physiology. The calibration result cannot by default be assumed to generalize beyond the validity conditions.

## Data Availability

The survey and the survey results (excluding professional experience and free-text options, to avoid potential identification) are publicly available (https://osf.io/gsb2e/). Full data are available from the corresponding author under a data protection agreement.

## Appendix: suggested task instructions

In this experiment, you will see different colored shapes on the screen. Please look at the screen at all times. You will also receive electric stimulations. There may or may not be a relationship between the colored shapes and electrical stimulations.

## Footnotes

1 One may reasonably use the term „learned“ in these cases, too. When we use the term „learned“ in the following, we tacitly imply „learned by direct experience“.
